# Genome‐wide alternative splicing profiling in the fungal plant pathogen *Sclerotinia sclerotiorum* during the colonization of diverse host families

**DOI:** 10.1111/mpp.13006

**Published:** 2020-10-28

**Authors:** Heba M.M. Ibrahim, Stefan Kusch, Marie Didelon, Sylvain Raffaele

**Affiliations:** ^1^ LIPM, Université de Toulouse INRAE CNRS Castanet‐Tolosan France; ^2^ Genetics Department Faculty of Agriculture Cairo University Giza Egypt; ^3^ Present address: Plant Health and Protection Division of Plant Biotechnics Department of Biosystems Faculty of Bioscience Engineering KU Leuven Leuven Belgium; ^4^ Present address: Unit of Plant Molecular Cell Biology Institute for Biology I RWTH Aachen University Aachen Germany

**Keywords:** alternative splicing, computational analysis, host adaptation, isoforms, RNA sequencing (RNA‐seq), *Sclerotinia sclerotiorum*

## Abstract

*Sclerotinia sclerotiorum* is a notorious generalist plant pathogen that threatens more than 600 host plants, including wild and cultivated species. The molecular bases underlying the broad compatibility of *S. sclerotiorum* with its hosts is not fully elucidated. In contrast to higher plants and animals, alternative splicing (AS) is not well studied in plant‐pathogenic fungi. AS is a common regulated cellular process that increases cell protein and RNA diversity. In this study, we annotated spliceosome genes in the genome of *S. sclerotiorum* and characterized their expression in vitro and during the colonization of six host species. Several spliceosome genes were differentially expressed in planta, suggesting that AS was altered during infection. Using stringent parameters, we identified 1,487 *S. sclerotiorum* genes differentially expressed in planta and exhibiting alternative transcripts. The most common AS events during the colonization of all plants were retained introns and the alternative 3′ receiver site. We identified *S. sclerotiorum* genes expressed in planta for which (a) the relative accumulation of alternative transcripts varies according to the host being colonized and (b) alternative transcripts harbour distinct protein domains. This notably included 42 genes encoding predicted secreted proteins showing high‐confidence AS events. This study indicates that AS events are taking place in the plant pathogenic fungus *S. sclerotiorum* during the colonization of host plants and could generate functional diversity in the repertoire of proteins secreted by *S. sclerotiorum* during infection.

## Graphical abstract

Alternative splicing occurs in the plant‐pathogenic fungus *Sclerotinia sclerotiorum* during colonization of diverse host plants and could generate functional diversity in the repertoire of secreted proteins.

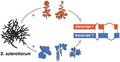

## 1 INTRODUCTION

*Sclerotinia sclerotiorum* is a plant‐parasitic fungus that causes white mould disease. It is known for its aggressive necrotrophic lifestyle, which means that the fungus actively kills the plant host cells and thrives by feeding on the dead plant material, and for exhibiting a broad host range. *S. sclerotiorum* can infect more than 600 host plants including economically important species such as tomato (*Solanum lycopersicum*), sunflower (*Helianthus annuus*), common bean (*Phaseolus vulgaris*), and beetroot (*Beta vulgaris*) (Boland and Hall, 1994; Peltier et al., 2012; Naito and Sugimoto, 1986).

To establish successful colonization of plants, *S. sclerotiorum* synthesizes and secretes oxalic acid, notably to establish favourable acidic conditions in host tissues (Liang and Rollins, 2018). Like its close relative *Botrytis cinerea*, *S. sclerotiorum* produces small RNAs suggested to interfere with the transcription of host defence genes (Derbyshire et al., 2019). In addition, *S. sclerotiorum* employs small secreted proteins to alter host cell physiology. A number of these have been characterized through mutant studies (Mbengue et al., 2016; Xia et al., 2019) and the release of the *S. sclerotiorum* genome sequence (Amselem et al., 2011; Derbyshire et al., 2017) enabled systematic searches for candidates (Guyon et al., 2014). Large‐scale studies of the *S. sclerotiorum* genome, proteome, and transcriptome also identified a large repertoire of secreted hydrolytic enzymes (Seifbarghi et al., 2017) produced during infection. Furthermore, *S. sclerotiorum* produces secondary metabolites such as sclerin and botcinic acid acting as toxins to facilitate infection (Graham‐Taylor et al., 2020; Pedras and Ahiahonu, 2004). Finally, *S. sclerotiorum* is able to detoxify plant defence compounds, enabling the colonization of certain hosts (Pedras and Ahiahonu, 2005), but the underlying molecular mechanisms are known in rare cases only (Chen et al., 2020). Whether the requirement for these diverse virulence mechanisms varies from one host to another remains largely elusive.

The coevolution of pathogen‐secreted proteins with their host targets (Dong et al., 2015) suggests that the ability to infect very diverse host species would associate with expanded repertoires of secreted proteins. However, the repertoire of secreted protein‐coding genes in *S. sclerotiorum* is within the average for ascomycete fungal pathogens (Derbyshire et al., 2017). To support infection of very diverse hosts, *S. sclerotiorum* exhibits codon usage optimization for secreted proteins, increasing the efficiency of protein translation with the potential to confer fitness benefits on multiple hosts (Badet et al., 2017). In addition, *S. sclerotiorum* hyphae organize in cooperating units, sharing the metabolic cost of virulence and growth during the colonization of resistant plants (Peyraud et al., 2019). Posttranscriptional regulation has been proposed as a mechanism to diversify effector proteins produced by a single gene (Betz et al., 2016), but support for this hypothesis remains scarce. Here, we investigated the extent to which posttranscriptional regulation could generate diversity in virulence factor candidates produced by *S. sclerotiorum* during the colonization of plants from diverse botanical families.

Alternative splicing (AS) is a process in eukaryotic cells that increases the cellular capacity to shape their transcriptome diversity and proteome complexity. Splicing is an important mechanism that regulates the maturation of the precursor messenger RNAs (pre‐mRNA) by subjecting it to the removal of noncoding sequences (introns). AS occurs in many eukaryotes under certain conditions, resulting in multiple isoforms of transcripts that retain specific intronic sequences or lack specific exonic sequences. The transcripts with retained introns (RI) then have a prolonged lifetime compared to the completely mature mRNA transcript (Braunschweig et al., 2014; Naro et al., 2017; Schmitz et al., 2017).

The efficiency and accuracy of the splicing mechanisms play a critical role in gene transcription and subsequent protein function. Imprecise splicing may result in abnormal and nonfunctional transcripts that may lead to the production of defective proteins, thus disturbing cellular processes. Previous studies showed that inaccurate splicing may cause diseases in humans, for example Parkinson's disease and leukaemia (David and Manley, 2010; Fu et al., 2013), and increases plant sensitivity to abiotic or biotic stresses (Cui et al., 2014). In line with this, the importance of AS in plant immunity against pathogen attacks is well established (Rigo et al., 2019). AS regulation and the factors that control it, the prediction of their *cis*‐regulatory sequences, and *trans*‐acting elements have been intensively studied in plants and in animals (Blanco and Bernabeu, 2011; Eckardt, 2013; Zhang et al., 2017), while only few reports are available from fungal phytopathogens. Therefore, the extent to which AS is regulated and functional during host colonization in fungal phytopathogens remains elusive.

Recently, Jin et al. (2017) found that transcripts of the plant fungal pathogen *Verticillium dahliae* undergo splicing of retained introns, producing different isoforms of transcripts. These isoforms have predicted roles in controlling many conserved biological functions, such as ATP synthesis and signal transduction. The involvement and regulation of the retained intron isoforms and splicing during the infection of host plants are still unexplored. Moreover, AS is detected during *V. dahliae* microsclerotia development (Xiong et al., 2014). Interestingly, 90% of the detected alternative transcripts exhibit retained introns. However, there is no further evidence to support the contribution of AS in microsclerotia development. In the same fashion, alternative transcripts are annotated in the genomes of the plant‐pathogenic fungi *Colletotrichum graminicola* and *Fusarium graminearum* (Schliebner et al., 2014; Zhao et al., 2013).

AS is pivotal in regulating gene expression and in diversification of the protein repertoire in the plant‐pathogenic oomycete *Pseudoperonospora cubensis* during pathogen development and transition from sporangia to zoospores (Burkhardt et al., 2015). In this study 4,205 out of 17,558 genes with c.10,000 potential AS events were identified, of which c.83% had evidence of retained introns. Interestingly, no exon skipping events were detected. Intriguingly, two genes encoding putative secreted RXLR and QXLR effectors showed evidence for a retained intron specifically at the sporangia stage, while the spliced version was abundant during the host‐associated stage. The retained intron may, therefore, regulate gene expression instead of affecting the function of the protein. Similarly, AS of the genes encoding glyceraldehyde‐3‐phosphate dehydrogenase (GAPDH) and 3‐phosphoglycerate kinase (PGK) modulates their localization in the smut fungus *Ustilago maydis*. In particular, AS gives rise to GAPDH carrying a peroxisome‐targeting signal. Importantly, *U. maydis* mutants lacking the specific isoforms with peroxisomal localization have reduced virulence (Freitag et al., 2012). These examples highlight the crucial role of AS in the pathogenicity of plant‐pathogenic fungi.

A predicted splicing factor 8 corresponding to the U5‐associated component Prp8 (GenBank accession number SS1G_03208) was reported recently from *S. sclerotiorum* (McLoughlin et al., 2018). This prompted us to test for AS in *S. sclerotiorum* during the infection of diverse host plants. To this end, we exploited RNA‐seq data of *S. sclerotiorum* infecting host plants from six botanical families, that is, *Arabidopsis thaliana* (Brassicales), tomato (Solanales), sunflower (Asterales), beetroot (Caryophyllales), castor bean (*Ricinus communis*, Malphigiales), and common bean (Fabales), in addition to the RNA‐seq of *S. sclerotiorum* cultivated in vitro as control (Peyraud et al., 2019; Sucher et al., 2020). We found that *S. sclerotiorum* has a functional splicing machinery and that at least 4% of the *S. sclerotiorum* secretome undergoes AS regulation, resulting in multiple differentially expressed isoforms that may have modified or altered functions. Some of the novel transcripts exhibit different predicted function or localization. Based on our analysis, we suggest that AS has the potential to give rise to transcriptional flexibility, thus contributing to the broad host spectrum of the plant‐pathogenic fungus *S. sclerotiorum*.

## 2 RESULTS

### 2.1 *S. sclerotiorum* spliceosome is differentially regulated during host colonization

**FIGURE 1:**
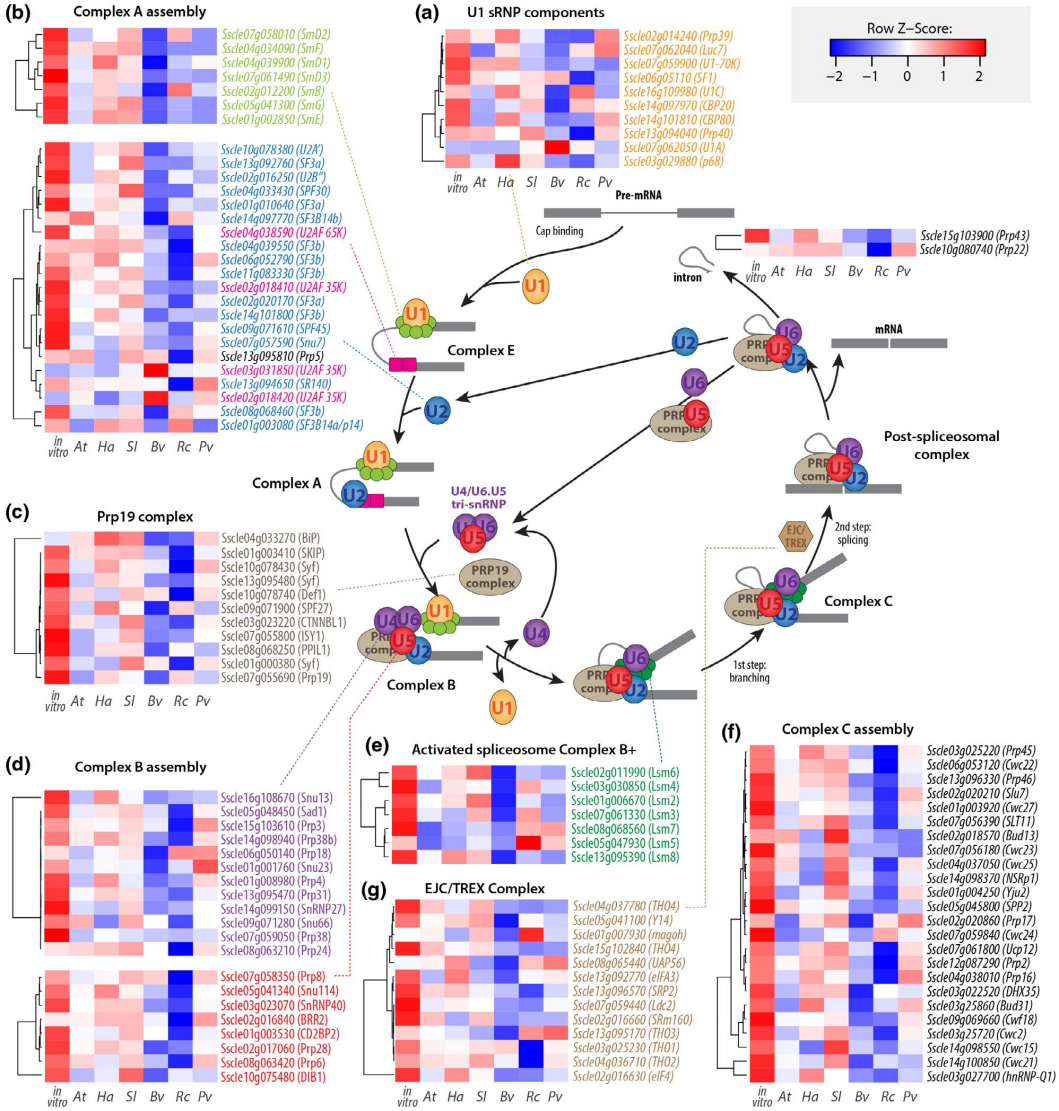
Identification of *Sclerotinia sclerotiorum* spliceosome components and their transcriptional regulation during the infection of plants from six botanical families. Diagrammatic representation of mRNA splicing process featuring *S. sclerotiorum* genes involved in each step. A hypothetical pre‐mRNA molecule is depicted with two exons shown as dark grey boxes and an intron shown as dark grey line. Circles, rounded rectangles, and hexagons show protein complexes. The relative gene expression for 116 spliceosome genes at the edge of *S. sclerotiorum* mycelium during infection of plants from six species and in vitro is shown as heatmaps. Pre‐mRNA splicing involves multiple spliceosomal complexes. First, complex E is established by binding of U1 snRNP (a) to small nuclear ribonucleoprotein‐associated proteins (Sm), U2 associated factors (U2AF), and splicing factors (SF), leading to the recruitment of U2 and the formation of complex A (b). The PRP19C/Prp19 complex/NTC/19 complex (c) stabilizes the U4/U5/U6 tri‐snRNP spliceosomal complex leading to complex B assembly (d). The U1/U4 snRNPs are released to form the activated spliceosome complex B+ (e) triggering branching, intron excision, conformational rearrangements into complex C (f) and ligation of the proximal and distal exons. EJC/TREX is recruited to spliced mRNAs to mediate export to the cytoplasm (g). *At*, *Arabidopsis thaliana*; *Bv*, *Beta vulgaris*; *Ha*, *Helianthus annuus*; *Pv*, *Phaseolus vulgaris*; *Rc*, *Ricinus communis*; *Sl*, *Solanum lycopersicum*

To study AS in the fungal plant pathogen *S. sclerotiorum*, we first searched the predicted proteome of *S. sclerotiorum* for components associated with splicing (spliceosome) using BLASTP and UniProtKB. We identified all the main components encompassing the entire pre‐mRNA splicing cycle, that is, U1/U2/U4/U5/U6‐associated components, PRP19/NTC complex proteins, the proteins catalysing the splicing of the intron (exon junction complex; EJC), the mRNA export complex TREX, and the mRNA and intron release components PRP43 and PRP22 (Figure 1).

We documented the transcriptional regulation of *S. sclerotiorum* spliceosome components during plant infection by exploiting RNA‐seq reads of *S. sclerotiorum* 1980 cultivated in vitro on potato dextrose agar (PDA) (Peyraud et al., 2019) and during the infection of host plants from six botanical families (Sucher et al., 2020): *A. thaliana* (At), tomato (*Solanum lycopersium*, Sl), sunflower (*Helianthus annuus*, Ha), common bean (*Phaseolus vulgaris*, Pv), castor bean (*Ricinus communis*, Rc), and beetroot (*Beta vulgaris*, Bv) (Figure 1). We found 116 proteins probably associated with (alternative) splicing, all but one of which were expressed at >10 fragments per kilobase of transcript per million mapped reads (FPKM) across all conditions (Figure 1 and Table S1). *Sscle02g018420*, encoding a U2AF, was not expressed at detectable levels (FPKM < 1). By performing BLASTP searches we identified 81 of these 116 spliceosome‐associated genes to be conserved in related ascomycetes, such as *Botrytis* species (Table S2). Interestingly, many components exhibited the strongest expression in vitro, but appeared to be down‐regulated on some or all of the hosts. Eighty of the 116 genes were significantly down‐regulated (*p* < .01) on at least one host plant, and one gene (*Sscle03g031850*, encoding a U2AF) was up‐regulated on all hosts except sunflower (Table S3). For example, 63 components were down‐regulated on *B. vulgaris*, while the U2AF‐encoding gene *Sscle03g031850* displayed 4.7‐fold up‐regulation during infection of *B. vulgaris* (Figure 1). Overall, 81 of the 116 components appeared to be differentially modulated dependent on the host plant, suggesting host plant‐specific regulation of the spliceosome in *S. sclerotiorum*.

### 2.2 Alternatively spliced genes are differentially expressed during host infection

**FIGURE 2:**
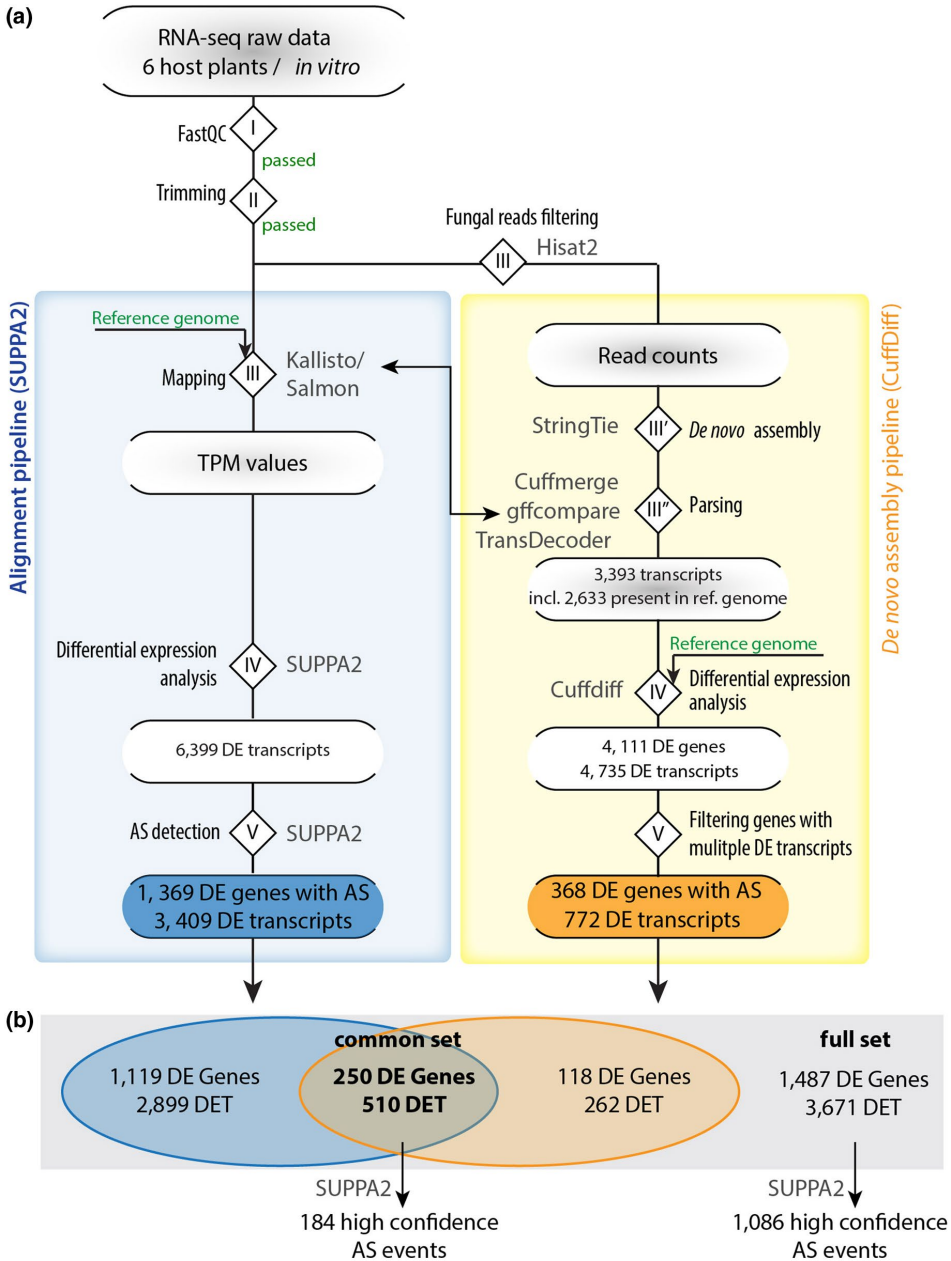
Pipeline for genome‐wide detection of alternative splicing (AS) in *Sclerotinia sclerotiorum*. (a) Raw RNA‐seq data was first inspected with FastQC (I) and quality‐trimmed using Trimmomatic (II). We then applied two pipelines for detection and analysis of novel transcripts, the de novo assembly pipeline (yellow box) and the transcriptome alignment pipeline (blue box). For detection of novel transcripts, we mapped reads with HISAT (III) to the *S. sclerotiorum* reference genome; this data was used in a modified StringTie de novo assembly (III’). Using Cuffmerge, gffcompare, and Transdecoder, we identified novel transcripts compared with the reference gene annotation (III’’) and generated a new reference annotation. Using the new annotation and the reference genome, we performed differential expression analysis (IV) and filtered the differentially expressed (DE) genes for those encoding at least two DE transcripts (DET; V). In the transcriptome alignment pipeline, mapping was done with Kallisto or Salmon (III), DE analysis and AS detection with SUPPA2 (IV and V). (b) A Venn diagram summarizing the results from DE analysis in (a) for both pipelines; numbers are given only for genes encoding multiple transcripts. AS, alternative splicing; DE, differentially expressed; DET, differentially expressed transcript; incl., including; ref., reference; TPM, transcripts per million

To search for AS events in the *S. sclerotiorum* transcriptome in planta and to reduce false discovery rate due to pipeline‐dependent bias, we applied a stringent strategy based on two pipelines employing either transcriptome alignment or de novo transcriptome assembly (Figure 2a). Transcriptome alignment is a robust and effective method of characterizing transcripts that are mapped to a provided reference transcriptome (including isoforms with skipped exons) while de novo transcriptome assembly mainly focuses on recovering transcripts with segments of the genome that are missing from the transcriptome alignment method, including retained introns (Martin and Wang, 2011).

In the transcriptome alignment pipeline (Figure 2a), the trimmed reads (steps I and II) were aligned to the *S. sclerotiorum* 1980 reference genome (Derbyshire et al., 2017) (step III). Expression of transcripts (transcripts per million, TPM) was determined in Salmon with the QUASI mapping algorithm (Patro et al., 2017) and Kallisto (Bray et al., 2016). Next, we used SUPPA2 to identify differentially expressed (DE) transcripts (step IV), with a cut‐off TPM > 30 and *p* < .05 (Trincado et al., 2018). We found 6,399 DE transcripts in total with this approach among all samples (lesion edge on six plant species) and compared to the control (edge *S. sclerotiorum* cultivated in vitro on PDA). Then, SUPPA2 was applied to DE genes to identify the different AS events and to measure the percentage spliced in index (PSI; ψ), which represents the ratio between reads excluding or including exons (step V). These PSI values indicate the inclusion of sequences into transcripts (Alamancos et al., 2015; Wang et al., 2008) using the normalized transcript abundance values (TPM) of the isoforms from Salmon. The differential splicing analysis of the events (dpsi values) at *p* < .05 identified 1,369 DE genes with significant splicing events producing 3,409 DE transcripts (Figure 2a,b).

In the de novo assembly pipeline, transcripts were assembled from fungal reads using StringTie. To identify fungal reads in our samples, the trimmed reads (step I and II) were aligned to the *S. sclerotiorum* 1980 reference genome (Derbyshire et al., 2017) using HISAT2 (Kim et al., 2015), yielding between 10,258,270 and 26,314,353 mapped reads per sample (Table S4) (step III). *S. sclerotiorum* reads were then used for de novo transcriptome assembly in a modified Tuxedo differential expression analysis pipeline (Trapnell et al., 2010, 2012). Because StringTie was proven to be a more accurate and improved transcript assembler and quantifier (Pertea et al., 2015, 2016), we used StringTie instead of cufflinks for the de novo assembly step (step III’ and III”). This resulted in 3,393 transcripts, including 2,633 transcripts from genes present in the reference transcriptome of *S. sclerotiorum* isolate 1980 (Derbyshire et al., 2017), 410 gene fusions and 337 novel genes encoding 350 transcripts. We compared FPKM expression values of original and novel transcripts and found similar distributions for the two sets. We therefore expect the rate of spurious transcripts to be limited and similar in the two sets of transcripts (Figure S1 and FPKM values in Table S5). Differential expression analysis on the complete transcriptome including both reference and novel transcripts with cuffdiff (step IV) identified 4,111 DE genes accounting for 4,735 DE transcripts on any of the six host species compared to the control. Out of those, there were 368 genes that encoded several DE transcripts each, producing 772 transcripts in total. These represent candidate genes harbouring AS in planta (step V).

Finally, we compared transcripts identified with the two pipelines and found a total number of 3,671 transcripts differentially expressed in planta in total, originating from 1,487 genes (“full set” of candidates). Among those, the two pipelines identified a common set of 250 genes of *S. sclerotiorum* encoding more than one transcript and expressed differentially in planta (“common set” of candidates, Figures 2b and S2). This common set of genes produced 510 transcripts differentially expressed in planta. To homogenize AS event predictions on these genes, we re‐ran SUPPA2 to calculate PSI values for genes from the common and full sets of candidates. This identified 1,086 high‐confidence AS events in the full set of genes and 184 high‐confidence AS events in the common set of genes.

### 2.3 The AS landscape in *S. sclerotiorum* during host colonization

**FIGURE 3:**
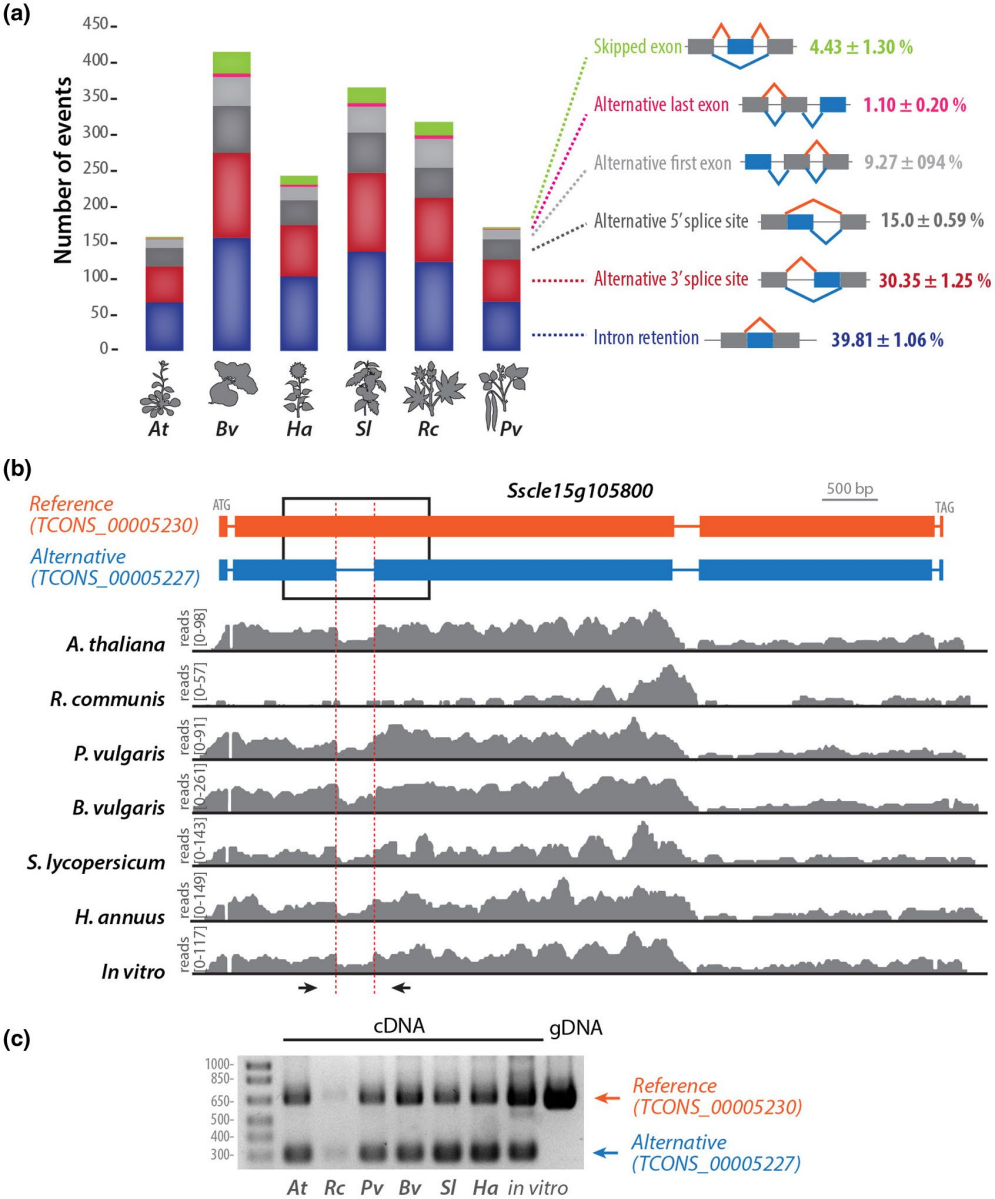
Intron retention is the major type of alternative splicing (AS) event in *Sclerotinia sclerotiorum* during the colonization of plants from diverse botanical families. (b) Distribution of high‐confidence AS events identified by SUPPA2 according to type of event and host plant infected by *S. sclerotiorum*. In diagrams depicting the different type of events, orange lines show intron splicing pattern in the reference transcript, blue lines show intron splicing pattern in the alternative transcript, blue boxes show alternatively spliced exons, grey boxes show invariant exons. Percentages indicate the relative proportion of one AS event type relative to all AS events identified during infection of a given plant species. (b) Example of an intron retention event in the reference transcript of *Sscle15g105800*. In the transcripts diagram, exons are shown as boxes, introns as lines. Read mappings are shown in grey for one RNA‐seq sample of each treatment. (c) Reverse transcription (RT)‐PCR analysis of *Sscle15g105800* transcripts produced during the colonization of six plant species and in vitro. The position of oligonucleotide primers used for RT‐PCR is shown as arrows in (b). *At*, *Arabidopsis thaliana*; *Bv*, *Beta vulgaris*; *Ha*, *Helianthus annuus*; *Pv*, *Phaseolus vulgaris*; *Rc*, *Ricinus communis*; *Sl*, *Solanum lycopersicum*

To document the effect of AS on *S. sclerotiorum* genes differentially expressed in planta, we performed AS events detection with SUPPA2 on genes induced on each plant and classified AS by type of event on each plant. The number of AS events varied 2.63‐fold according to host, ranging from 158 AS events in *A. thaliana* to 415 AS events in *B. vulgaris*, reaching a total 1,086 distinct AS events for the six plant species (Figure 3a). The distribution of AS event type did not differ significantly during colonization of the six different host plants (Figure 3a). Retained intron was the major type of AS event detected in *S. sclerotiorum* during host colonization (RI; 39.8 ± 1.1%), followed by alternative 3′ receiver site (A3; 30.3 ± 1.2%), alternative 5′ donor site (A5;15.0 ± 06%), skipped exon (SE; 4.4 ± 1.3%), and alternative first exon (AF; 1.1 ± 0.2%).

In all hosts, the most frequent AS event was intron retention, of which the gene *Sscle15g105800* is one example. This gene belongs to the full set of AS gene candidates and encodes a 2,043 amino acid long predicted protein of unknown function conserved in *B. cinerea*. StringTie identified for this gene a transcript TCONS_00005227 with five exons and four introns (Figure 3b). The reference transcript TCONS_00005230 harbours four exons, including a 3,958 bp exon 2 corresponding to the fusion between TCONS_0005227 exon 2, retained intron 2 (351 bp), and exon 3. Reads aligned to TCONS_0005227 intron 2 were detected in all RNA‐seq samples and were particularly abundant during infection of *A. thaliana*, *B. vulgaris*, and *P. vulgaris*. *Sscle15g105800* was weakly expressed on *R. communis* with few reads aligned to TCONS_0005227 intron 2 (Figure 3b). To confirm AS of TCONS_0005227 intron 2, we performed reverse transcription (RT)‐PCR with primers spanning this intron on genomic DNA and on cDNAs produced from *S. sclerotiorum* grown in vitro (PDA) and infected plants (Figure 3c). Amplicons from the reference transcript TCONS_00005230 were detected on all cDNAs, albeit only weakly on cDNAs produced from infected *R. communis*. An alternative transcript was detected on all cDNAs, the size of which corresponds to TCONS_00005227 retained intron 2.

### 2.4 AS is host‐regulated in *S. sclerotiorum*

**FIGURE 4:**
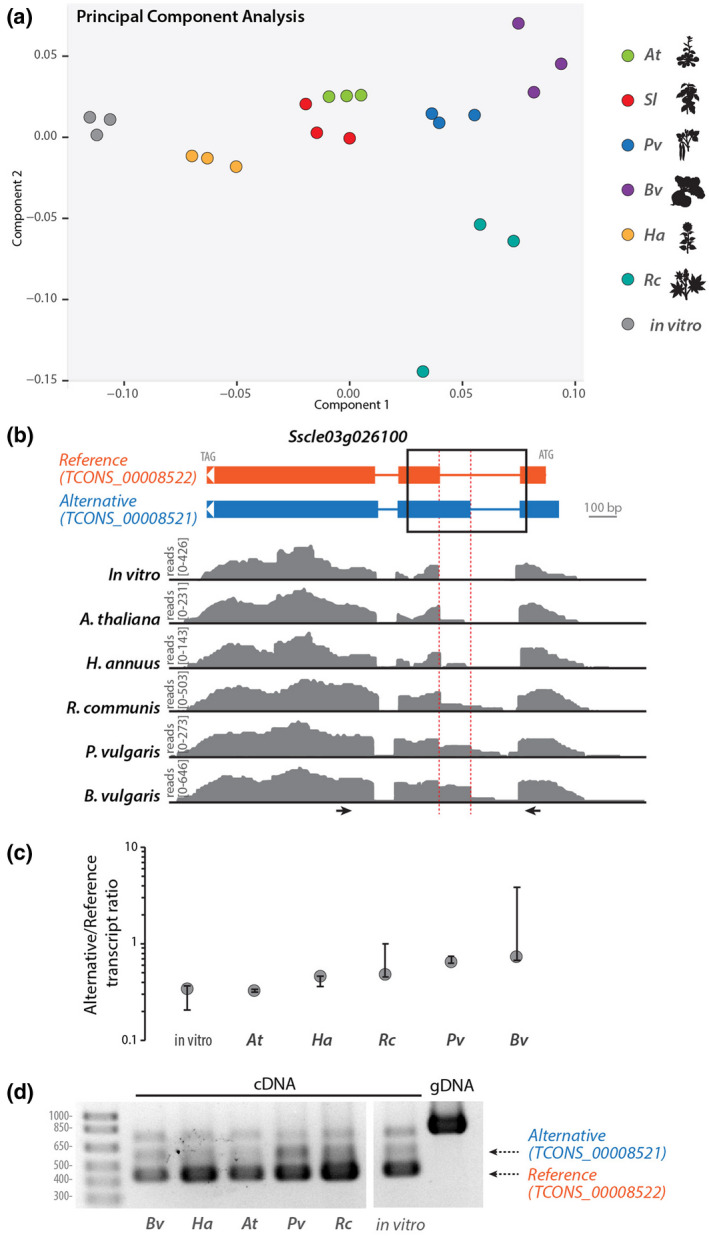
Alternative 3′ receiver splice site variation according to host in *Sscle03g026100*. (a) Principal component analysis map of the sample variable for the accumulation of reference and alternative transcripts produced by 250 high‐confidence *S. sclerotiorum* genes showing alternative splicing (AS). Sample types are colour‐coded according to infected host plant species. (b) Example of alternative 3′ receiver splice site in the reference transcript of *Sscle03g026100*. In the transcript diagrams, exons are shown as boxes, introns as lines. Read mappings are shown in grey for one RNA‐seq sample of each treatment. (c) Ratio between the abundance of alternative over reference transcript for *Sscle03g026100* determined by Cuffdiff. Error bars show 90% confidence interval. (d) Reverse transcription (RT)‐PCR analysis of *Sscle03g026100* transcripts produced during the colonization of five plant species and in vitro. The position of oligonucleotide primers used for RT‐PCR is shown as arrows in (b). *At*, *Arabidopsis thaliana*; *Bv*, *Beta vulgaris*; *Ha*, *Helianthus annuus*; *Pv*, *Phaseolus vulgaris*; *Rc*, *Ricinus communis*; *Sl*, *Solanum lycopersicum*

To document the extent to which host plant species associated with AS events in *S. sclerotiorum*, we performed hierarchical clustering and principal component analysis (PCA) for *S. sclerotiorum* alternatively spliced transcript accumulation in six host species (Figure 4a). The distribution of the plant variable according to the two principal components displayed host‐specific clustering in which the AS transcripts produced on each host could be clearly separated, except for AS transcripts produced on *A. thaliana* and *S. lycopersicum*. This analysis suggested that the relative accumulation of alternative transcripts produced by a given gene could vary according to the host being colonized.

We tested whether this was the case for the gene *Sscle03g026100*, encoding a predicted phosphoenolpyruvate kinase‐like protein. The *Sscle03g026100* locus harboured RNA‐seq reads that aligned in the 3′ region of intron 1, indicative of alternative 3′ receiver sites in exon 2 of the reference transcripts (*TCONS_00008522*, Figure 4b). This splicing event is predicted to cause an extension of the alternatively spliced exon in transcript variant *TCONS_00008521*. Thanks to its N‐terminal extension, the protein isoform TCONS_0008522 but not TCONS_00008521 is recognized as a member of the PIRSF034452 family (TIM‐barrel signal transduction protein). According to Cuffdiff transcript quantification, the ratio between alternative and reference transcript varied from 0.32 in *A. thaliana* to 0.73 in *B. vulgaris* (Figure 4c). To confirm AS of the *Sscle03g026100* transcript, we performed RT‐PCR with primers spanning the variant exon 2 on RNAs collected from five host species (Figure 4d). We retrieved a 418 bp amplicon corresponding to the reference transcript (*TCONS_00008522*), a 535 bp amplicon corresponding to the *TCONS_00008521* alternative transcript, as well as a third c.750 bp amplicon. In agreement with the RNA‐seq read coverage, bands corresponding to the alternative transcript *TCONS_0008521* were much weaker than bands corresponding to the reference transcript *TCONS_0008522* in *A. thaliana*, *H. annuus*, and in vitro.

### 2.5 AS is predicted to generate protein isoforms with modified functions

To study the functional consequences of AS in *S. sclerotiorum*, we first analysed gene ontology (GO) terms enriched in our list of high‐confidence DE genes with AS. GO enrichment was determined using BiNGO, a tool package within the complex network visualizing platform Cytoscape (Shannon et al., 2003; Maere et al., 2005) (Table S6). The most significantly enriched terms included “oxidoreductase activity” and “carbohydrate metabolic process”, suggesting that genes involved in the degradation of carbohydrates and organic molecules were subject to AS during infection of host plants. According to BLASTP searches (*E* < 10^−25^) 175 of the 250 genes in our common set of AS candidates are conserved in related ascomycetes, including *Botrytis* species (Table S7).

Second, to test if AS could alter the domain content of protein isoforms in our full set of AS candidates, we assigned PFAM domains to all isoforms and identified AS events leading to a change in PFAM domain content. In total, 158 genes expressed alternative transcripts with changes in PFAM annotation profiles. Of these, 53 isoforms exhibited loss of PFAM domains, 85 isoforms displayed gain of PFAM domains, and 20 isoforms showed more complex changes of PFAM profiles (File S1). Only eight of the 158 genes with alterations in their PFAM annotation profiles were from the 250 common AS genes (Table 1). Four isoforms gained PFAM domains, for example the putative cutinase Sscle11g080920 where the alternative isoform TCONS_00002255 gained two PFAM domains, ETS_PEA3_N (PF04621.12) and CBM_1 (PF00734.17).

**TABLE 1:** Changes in PFAM profiles of differentially expressed and alternatively spliced genes of *Sclerotinia sclerotiorum*

Gene	Isoform	PFAM accessions	PFAM descriptors
Sscle02g012330	TCONS_00006935	PF05277	DUF726
	TCONS_00006936	PF04900; PF05277	Fcf1; DUF726
	TCONS_00006937	PF04900; PF09388	Fcf1; SpoOE‐like
Sscle03g026280	TCONS_00008535	PF00172; PF05393; PF07690; PF08006; PF14960	Zn_clus; Hum_adeno_E3A; MFS_1; DUF1700; ATP_synth_reg
	TCONS_00008536	PF00172; PF05393; PF07690; PF08006; PF14960; PF04082	Zn_clus; Hum_adeno_E3A; MFS_1; DUF1700; ATP_synth_reg; Fungal_trans
	TCONS_00008537	PF00172	Zn_clus
Sscle03g030170	TCONS_00008807	PF06999	Suc_Fer‐like
	TCONS_00008808		
Sscle03g031900	TCONS_00008259	PF01601	Corona_S2
	TCONS_00008260		
Sscle05g043820	TCONS_00010732	PF06172	Cupin_5
	TCONS_00010733		
Sscle11g080920	TCONS_00002254	PF01083; PF08237	Cutinase; PE‐PPE
	TCONS_00002255	PF01083; PF08237; PF04621; PF00734	Cutinase; PE‐PPE; ETS_PEA3_N; CBM_1
Sscle15g103140	TCONS_00005366	PF00169; PF01442; PF11932	PH; Apolipoprotein; DUF3450
	TCONS_00005367	PF00169; PF01442; PF11932; PF05592; PF17389; PF17390	PH; Apolipoprotein; DUF3450; Bac_rhamnosid; Bac_rhamnosid6H; Bac_rhamnosid_C
	TCONS_00005368	PF00169; PF01442; PF11932	PH; Apolipoprotein; DUF3450
Sscle16g109930	TCONS_00006105	PF00032; PF00083; PF03137; PF07690; PF12670	Cytochrom_B_C; Sugar_tr; OATP; MFS_1; DUF3792
	TCONS_00006106	PF00032; PF00083; PF03137; PF07690; PF12670; PF05977	Cytochrom_B_C; Sugar_tr; OATP; MFS_1; DUF3793; MFS_3

**FIGURE 5:**
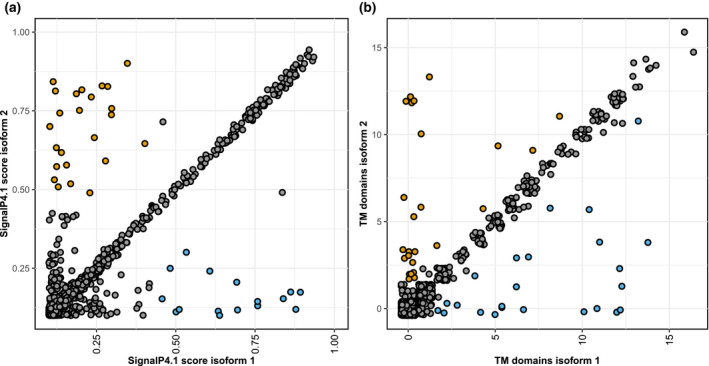
Alternative splicing (AS) modifies secretion potential of proteins in *Sclerotinia sclerotiorum*. (a) We determined the likelihood of presence of an N‐terminal secretion signal using SignalP v. 4.1 in the protein encoded by the reference transcript (isoform 1) and the alternative transcripts (isoform 2). The scatterplot shows the SignalP scores of both isoforms for all alternatively spliced genes of *S. sclerotiorum*, where a score of 0.45 is the threshold for a putative secretion peptide. Orange data points indicate novel isoforms that may have gained a secretion peptide, blue data points indicate loss of the secretion peptide. (b) We predicted the number of transmembrane domains for reference (isoform 1) and novel isoforms (isoform 2) using TMHMM. Orange data points indicate isoforms that gained transmembrane domains, blue data points indicate novel isoforms that may have lost one or more transmembrane domains

Furthermore, we explored the isoforms from alternatively spliced genes for signal peptides for secretion (Figure 5a). Of the 250 genes in our common set of AS candidates, 42 are predicted to encode a secreted protein, corresponding to 4% of the *S. sclerotiorum* secretome (Juan et al., 2019; Figure S3). Among those, five genes (*Sscle02g014060*, *Sscle07g057820*, *Sscle09g070580*, *Sscle12g091110*, and *Sscle15g103140*) showed possible gains of secretion peptide by AS and two cases of loss of secretion peptides in alternative isoforms (*Sscle10g075480* and *Sscle15g102380*). In the set of 3,393 AS candidates detected in total, we found 26 possible gains of secretion peptides and 16 losses of secretion peptides in alternative isoforms.

Similarly, AS caused the gain and loss of transmembrane domains in novel isoforms (Figure 5b). In total, 20 novel isoforms exhibited gain of one and 25 isoforms gained more than one predicted transmembrane domains. Thirty‐two of these did not harbour a putative transmembrane domain in the reference isoform, which suggests relocalization to the plasma membrane or an intracellular membrane. Conversely, we observed the loss of one transmembrane domain in 30 isoforms and of more than one in 24 isoforms, including 32 isoforms that lost all transmembrane domains, suggesting subcellular relocalization of the respective novel isoform. Two genes that gained (*Sscle05g040780* and *Sscle16g109930*) and five genes that lost (*Sscle02g014060*, *Sscle02g019060*, *Sscle03g031900*, *Sscle05g043820*, and *Sscle08g066940*) transmembrane domains are found in the 250 alternatively spliced and differentially expressed genes. Intriguingly, the novel isoform of Sscle02g014060 is predicted to be secreted as well as to have lost its transmembrane domain.

### 2.6 AS is predicted to modify the activity of *S. sclerotiorum* secreted proteins

**FIGURE 6:**
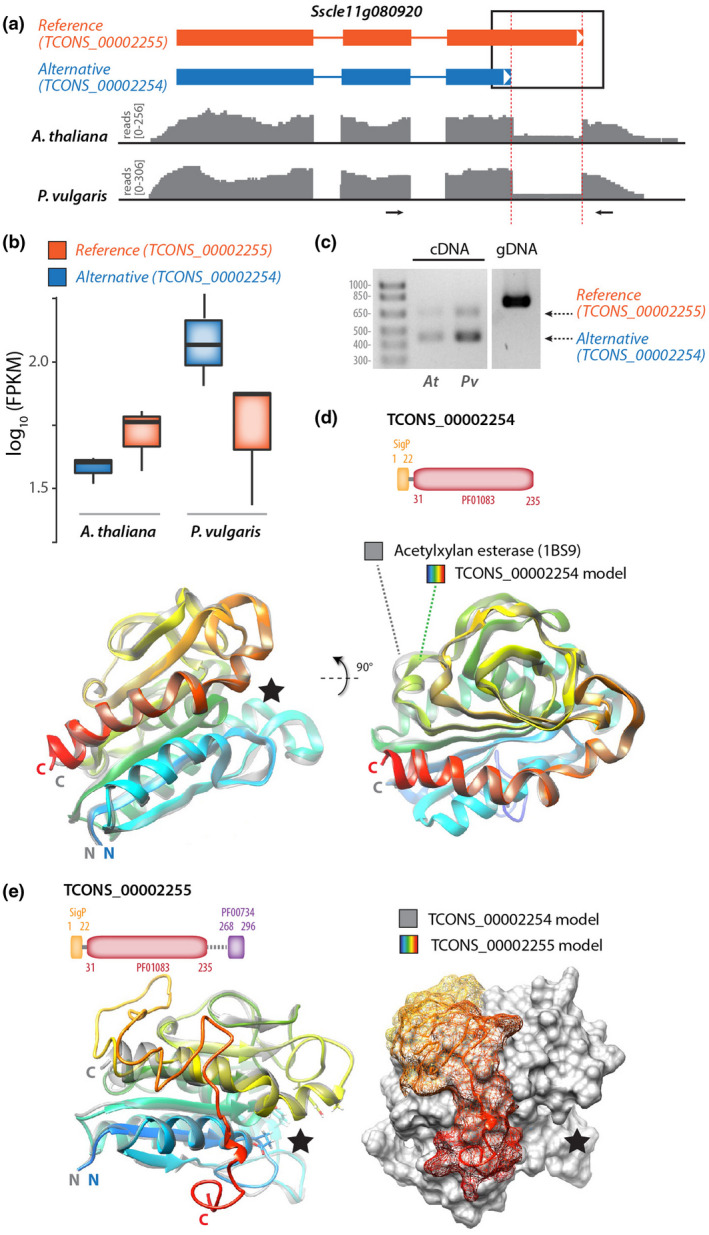
Alternative splicing (AS) generates structural diversity in the predicted secreted protein Sscle11g080920. (a) Consequences of alternative 5′ donor splice site in exon 3 of the reference transcript of *Sscle11g080920*. In the transcript diagrams, exons are shown as boxes, introns as lines. Read mappings are shown in grey for one RNA‐seq sample collected on infected *Arabidopsis thaliana* and infected *Phaseolus vulgaris*. (b) Relative accumulation of the reference and alternative transcripts produced by *Sscle11g080920* on infected *A. thaliana* and infected *P. vulgaris*. Boxplots show the expression of the transcripts TCONS_00002254 and TCONS_00002255 from RNA‐seq of *S. sclerotiorum* infecting *A. thaliana* and *P. vulgaris* in log_10_(FPKM). Boxplots show the median of the data points, whiskers are at 1.5 × interquartile range of the highest/lowest value. (c) Reverse transcription (RT)‐PCR analysis of *Sscle11g080920* transcripts produced during the colonization of *A. thaliana* (*At*) and *P. vulgaris* (*Pv*). The position of oligonucleotide primers used for RT‐PCR is shown as arrows in (a). (d) Diagram of the domain structure for the protein produced by TCONS_00002254 alternative transcript and ribbon model of TCONS_00002254 predicted protein structure (rainbow colours). TCONS_00002254 protein model is shown superimposed with its closest structural analog AXEII acetylxylan esterase (grey). The black star indicates the position of the active site in AXEII. (e) Diagram of the domain structure for the protein produced by TCONS_00002255 reference transcript and ribbon model of TCONS_00002255 predicted protein structure (rainbow colours). TCONS_00002255 protein model is shown superimposed with TCONS_00002254 model (grey). The black star indicates the position of the active site deduced from the analysis shown in (d)

Of the 250 genes with evidence for AS, 42 are predicted to encode a secreted protein, corresponding to 4% of the *S. sclerotiorum* secretome (Juan et al., 2019; Figure S3). For example, the alternatively spliced gene *Sscle11g080920* was predicted to encode two secreted protein isoforms derived from the reference transcript TCONS_00002255 and the alternative transcript TCONS_00002254, exhibiting an alternative 5′ donor splice site in exon 3 (Figure 6a). The relative accumulation of these two isoforms varied according to the plant being colonized. The reference transcript was expressed more strongly than the alternative transcript in *S. sclerotiorum* infecting *A. thaliana* (Figure 6b). In contrast, we measured higher accumulation of the alternative than the reference transcript in *S. sclerotiorum* infecting *P. vulgaris*. To confirm AS of the *Sscle11g080920* transcript, we performed RT‐PCR with primers spanning the variant exon 3 on RNA collected from *A. thaliana* and *P. vulgaris* (Figure 6c). As expected, this identified a 618 bp amplicon corresponding to the reference transcript and a 430 bp amplicon corresponding to the alternative transcript during the colonization of *A. thaliana* and *P. vulgaris*. In this assay, the alternative transcript accumulated more than the reference transcript during the infection of *P. vulgaris*, and to a lesser extent during the colonization of *A. thaliana*.

To gain insights into the functional consequence of AS in *Sscle11g080920*, we analysed PFAM domains and performed structure modelling for the proteins encoded by the reference transcript TCONS_00002255 and the alternative transcript TCONS_00002254. The alternative transcript TCONS_00002254 encoded a 235 amino acid protein featuring a secretion signal and a cutinase (PF01083) domain (Figure 6d). Homology modelling and fold recognition in I‐TASSER identified the acetylxylan esterase AXEII from *Penicillium purpureogenum* (PDB identifier 1BS9) as the closest structural analog to TCONS_00002254 (RMSD 0.31Å). AXEII is a close structural analog of *Fusarium solani* cutinase, an esterase that hydrolyses cutin in the plant's cuticle (Ghosh et al., 2001). The reference transcript TCONS_00002255 encoded a 296 amino acid protein featuring a secretion signal, a cutinase domain, and a short fungal cellulose‐binding domain (PF00734) (Figure 6e). Its closest structural analog identified by I‐TASSER was model 1G66 of AXEII. The superimposition of TCONS_00002254 and TCONS_00002255 protein models revealed that the C‐terminal extension in TCONS_00002255 corresponds to a surface‐exposed unstructured loop reaching the neighbourhood of the catalytic site cleft (Figure 6e). This additional exposed loop could modify protein–protein interactions in TCONS_00002255 or modify access to its catalytic site. These results suggest that AS is a mechanism to generate functional diversity in the repertoire of proteins secreted by *S. sclerotiorum* during the colonization of host plants.

## 3 DISCUSSION

There are several approaches to study AS from RNA‐seq data (Thakur et al., 2019), such as analysing splice junctions (Hu et al., 2013) or exonic regions (Anders et al., 2012), which largely rely on mapping strategies only. The pipeline we used in this study combines two fundamentally different strategies (de novo assembly based and reference mapping‐based) to detect true novel splicing events and reduce algorithm bias. This approach, however, does not completely exclude false‐positive or false‐negative AS events, and also does not allow a distinction between an AS event and correction of an incorrect reference gene model. Manual inspection or curation of gene models, as, for example, performed in *F. graminearum* (Zhao et al., 2013), is required to distinguish between these possibilities. In the current study, we limited our analysis to one stage of infection per host species, selected to represent similar infection stages on each host (Sucher et al., 2020). A time‐course experiment as, for example, performed for *Brassica napus* (Seifbarghi et al., 2017) would be likely to reveal additional biologically relevant isoforms that could support a specific stage of infection or host defence suppression. We have inspected AS predictions and experimentally validated alternative transcripts for a small subset of the AS events predicted here, supporting the accuracy of our analysis pipeline. Nevertheless, further efforts will be needed to improve the gene annotations of *S. sclerotiorum*, confirm alternative transcripts across all stages of infection, and identify further alternatively spliced transcripts missed by our pipeline at the genome scale.

### 3.1 A number of *S. sclerotiorum* genes are spliced alternatively on different hosts

Colonization of a host plant by a pathogen requires global changes in the gene expression of the pathogen and secretion of effector proteins and enzymes (van der Does and Rep, 2017). AS is a regulatory mechanism affecting the activity of a majority of genes in plant and animal cells at the posttranscriptional level. Whether AS contributes to the regulation of virulence in plant‐pathogenic fungi remains elusive. In this study, we present a comparative genome‐wide survey of AS in the plant‐pathogenic fungus *S. sclerotiorum* during the infection of six different host plants compared to growth in vitro as a control. Using stringent criteria for the detection of alternatively spliced isoforms, and considering genes identified consistently with our two pipelines, we found 250 genes that expressed more than one isoform (Figure 2). These represent about 2.3% of the genome, which is consistent with estimates for the AS rate of 2.7% in the closely related fungal species *B. cinerea* (Grützmann et al., 2014). In *F. graminearum,* which causes head blight disease in cereal and stalk rot in maize, AS represents 1.7% of the total number of genes in mycelia grown in vitro (Zhao et al., 2013), while in *C. graminicola*, which causes anthracnose disease in maize, only 0.57% of all genes are predicted to exhibit AS during maize infection (Schliebner et al., 2014). Yet this percentage is strikingly low compared to the AS rate of the intron or multiexon‐containing genes in plants such as *A. thaliana* or mammals such as *Homo sapiens*, which are reported to be 42% and 95%, respectively (Pan et al., 2008; Filichkin et al., 2010). AS is not well characterized in plant‐pathogenic fungi and needs to be investigated in more detail (Grützmann et al., 2014). A previous study reported evidence for AS in the plant‐pathogenic oomycete *Pseudoperonospora cubensis* that causes downy mildew in the Cucurbitaceae family (Burkhardt et al., 2015). In this work, 24% of the expressed genes showed novel isoforms with new AS events over the course of infection of cucumber at 1–8 days after infection. Moreover, recently Jin et al. (2017) found that the transcripts of two different isolates of the plant fungal pathogen *V. dahliae* undergo splicing of retained introns, producing different isoforms of transcripts that differ between the two isolates during the fungal development. These isoforms have predicted roles in controlling many conserved biological functions, such as ATP synthesis and signal transduction. Interestingly, *P. cubensis* exhibited 10‐fold higher AS rates than what we observed in *S. sclerotiorum* and in contrast to *S. sclerotiorum* is an obligate biotrophic pathogen. It is tempting to speculate that the intricate interaction of a biotrophic pathogen with its host plant requires an even more flexible and refined transcriptome than in nonpathogenic, hemibiotrophic, or necrotrophic fungi. Unfortunately, systematic studies addressing the question of the role of AS in the lifestyle of plant‐pathogenic fungi are lacking. Current research is limited to necrotrophic and hemibiotrophic fungi with a range of 2.3% (*S. sclerotiorum*) and 7.9% (the hemibiotrophic pathogen *Magnaporthe oryzae*) AS rate (Grützmann et al., 2014). Further studies should be conducted to uncover the contribution of AS to fungal lifestyles.

In our analysis, most of the AS events were retained introns (RI; 39.8%), which is consistent with previous studies where intron retention showed higher preference in the newly identified isoforms (Grützmann et al., 2014). Moreover, skipped exons represented a small frequency in our analysis (4.4%) but could be considered higher than usual compared to other fungi such as *V. dahliae* (2‐fold higher; 2.2%). Interestingly, SE is the most common AS event in mammals (Sammeth et al., 2008).

### 3.2 Do these AS variants contribute to virulence on the respective hosts?

AS is a natural phenomenon in eukaryotes that is genetically tightly regulated, and proper spliceosome activity ensures adequate splicing (Chen et al., 2012). The operating mechanisms of splicing regulation and the extent to which components of the splicing machinery regulate splice site decisions remain poorly understood, however (Saltzman et al., 2011). The spliceosome activity is modulated by *cis*‐ and *trans*‐acting regulatory factors. The *trans*‐acting elements include the SR (serine/arginine‐rich) and hnRNP (heterogeneous ribonucleoprotein) families (Chen and Manley, 2009; Nilsen and Graveley, 2010), and generally regulate AS by enhancing or inhibiting the assembly of the spliceosome at adjacent splice sites after perceiving *cis*‐acting elements in exon or intron regions of pre‐mRNAs.

Because spliceosome components strictly regulate splicing, any changes in spliceosome component abundance may result in inaccurate splicing and/or generation of alternative transcripts in accordance with the environmental condition that causes the changes. Although tightly regulated, AS is influenced by external stimuli in eukaryotes such as biotic and abiotic stresses. For instance, the LSM2–8 complex and SmE, which are regulatory components of the spliceosome, differentially modulate adaptation in response to abiotic stress conditions in *Arabidopsis* (Carrasco‐López et al., 2017; Huertas et al., 2019). Similarly, U1A is essential in adapting *Arabidopsis* plants to salt stress. Mutation in AtU1A renders *Arabidopsis* plants hypersensitive to salt stress and results in reactive oxygen species (ROS) accumulation (Gu et al., 2018).

In our analysis we found that many of the spliceosome components are down‐regulated in *S. sclerotiorum* during infection, in particular on hosts where we detected a high number of AS events. For instance, during infection of *B. vulgaris*, *S. sclerotiorum* exhibited the highest abundance of alternative transcripts and showed down‐regulation of the majority of the spliceosome components (Figures 1 and 3). This suggests that AS in *S. sclerotiorum* could result from the down‐regulation of spliceosome genes and raises the question of whether host plant defences actively interfere with the regulation of *S. sclerotiorum* spliceosomal machinery to trigger the observed down‐regulation. A related goal for future research will be determining whether AS confers fitness benefits to *S. sclerotiorum* during host colonization. Dedicated functional analyses will be required to clarify the role of AS in *S. sclerotiorum* adaptation to the host.

In some cases, more than one isoform is present in a host plant. The reason could be that the new isoforms have new functions that assist the establishment of pathogenesis, while the dominant isoform(s) has/have substantial biological functions that are needed for *Sclerotinia* under any condition. The different isoforms produced by AS in *S. sclerotiorum* during infection of the different host plants could be a way to increase pathogen virulence. For instance, the alternatively spliced gene *Sscle03g026100* encodes a putative phosphonopyruvate hydrolase. Phosphonopyruvate hydrolases hydrolyse phosphonopyruvate (P‐pyr) into pyruvate and phosphate (Liu et al., 2004). In plants, phosphonopyruvate plays an important intermediate role in the formation of organophosphonates, which function as antibiotics and play a role in pathogenesis or signalling. Therefore, the fungus may use these two different isoforms to detoxify one of the plant defence molecules to facilitate the infection process. In a previous study, *Ochrobactrumanthropi* and *Achromobacter* bacterial strains were found to degrade the organophosphates from surrounding environments and to use the degraded product as a source of carbon and nitrogen (Ermakova et al., 2017). Interestingly, the newly identified isoform of Sscle03g026100 (TCONS_00008521) showed the highest expression during infection of beans (*B. vulgaris*). Because *B. vulgaris* is well known for its production of antifungal secondary metabolites such as C‐glycosyl flavonoids and betalains (Citores et al., 2016; Ninfali et al., 2017), this suggests that the new isoform may be required for *S. sclerotiorum* to overcome the plant resistance by degrading some of these metabolites. In addition, AS of *Sscle11g080920*, predicted to encode a secreted cutinase, could exhibit specificity for differently branched cellulose molecules. Taken together, our study revealed that *S. sclerotiorum* uses AS that gives rise to functionally divergent proteins. We further show that a number of these isoforms have differential expression on diverse host plants.

## 4 EXPERIMENTAL PROCEDURES

### 4.1 Plant inoculations and RNA sequencing

Raw RNA‐seq data used in this work are available from the NCBI Gene Expression Omnibus under accession numbers GSE106811, GSE116194, and GSE138039. Samples and RNAs were prepared as described in Sucher et al. (2020). Briefly, the edge of 25 mm‐wide developed necrotic lesions were isolated with a scalpel blade and immediately frozen in liquid nitrogen. Samples were harvested before lesions reached 25 mm width, at 24 hr (*H. annuus*), 47–50 hr (*A. thaliana*, *P. vulgaris*, *R. communis*, and *S. lycopersicum*) or 72 hr postinoculation (*B. vulgaris*). Material obtained from leaves of three plants were pooled together for each sample, all samples were collected in triplicates. RNA extractions were performed using NucleoSpin RNA extraction kits (Macherey‐Nagel) following the manufacturer's instructions. RNA sequencing was outsourced to Fasteris SA to produce Illumina single‐end reads (*A. thaliana*, *S. lycopersicum*, in vitro control) or paired reads (other infected plants) using a HiSeq 2500 instrument.

### 4.2 Quantification of isoform and transcript abundance

Quality control for the RNA‐seq data was performed using FastQC (Babraham Bioinformatics). The quality‐checked data were processed for trimming with the Java‐based tool Trimmomatic‐0.36 (Bolger et al., 2014). Transcript abundances were quantified using a set of tools as follows. In the alignment pipeline, reads were first mapped to the *S. sclerotiorum* reference genome (Derbyshire et al., 2017) using HISAT2 (Kim et al., 2015). Annotation of reference genes and transcripts were provided in the input. The aligned reads were assembled and the transcripts were quantified in each sample using StringTie (Pertea et al., 2015, 2016). The assemblies produced by StringTie were merged with the reference annotation file in one GTF file to incorporate the novel isoforms with the original ones using cuffmerge (Goff et al., 2019). The accuracy of the merged assembly was estimated by reciprocal comparison to the *S. sclerotiorum* reference annotation. In the de novo assembly pipeline, transcript abundances were quantified using gffcompare (Pertea et al., 2016) and cuffcompare (Trapnell et al., 2012). All the expressed transcripts, including novel genes and alternatively spliced transcripts, were merged into one annotation file using the Tuxedo pipeline merging tool, cuffmerge. The accuracy of the assembled annotation file was assessed by comparing it to the reference genome using gffcompare (Pertea et al., 2016).

### 4.3 Differential expression analysis of RNA‐seq

The differential expression analyses of genes and isoforms were calculated using cuffdiff from the Tuxedo pipeline (Trapnell et al., 2012). We then used CummeRbund to visualize the cuffdiff results of the genes whose expression were marked as significant and at log_2_ fold change of ±2 across all samples, leaving 4,111 genes that had differentially expressed isoforms (Figure 2). *quasi‐mapping* was applied on the same RNA‐seq data for expression quantification of transcripts using Kallisto (Bray et al., 2016) and Salmon‐0.7.0 (Patro et al., 2017). Differential expression analysis of the quantified transcript and isoform abundance of the RNA‐seq data resulting from StringTie and Salmon were used in cuffdiff (Trapnell et al., 2012) and SUPPA2 (Trincado et al., 2018), respectively, according to the default parameters as referred to by the software manuals. The R Studio software package CummeRbund (Goff et al., 2019) was employed to determine the significant change in the transcript abundance across the different samples. All samples were compared with the PDA in vitro cultivation control. Default settings were used. Genes with a false discovery rate (FDR)‐adjusted *P* (*q*) < .05 with a fold change of ±2 were considered differentially expressed.

### 4.4 RNA‐seq data visualization and transcripts annotation

The Integrative Genomics Viewer (IGV) (Robinson et al., 2017) and WebApollo annotator (Lee et al., 2013) were used for visualizing the RNA‐seq data. Heatmaps were generated with the heatmap.2 function of R (R Core Team 2018). Spliceosome genes were identified using several approaches. A first set of genes were identified based on map 03040 (Spliceosome) for *S. sclerotiorum* (organism code “ssl”) in the Kyoto Encyclopedia of Genes and Genomes (KEGG). The annotation of these genes was verified using BLASTP searches against *Saccharomyces cerevisiae* and *H. sapiens* in the NCBI ReSeq database followed by searches in the UniprotKB database for detailed annotation. Second, we searched for all spliceosome components annotated in ascomycete genomes in the UniprotKB database and identified their orthologs in *S. sclerotiorum* using BLASTP searches. The gene ontology classification database with the Blast2GO package was used to perform the functional clustering of the differentially expressed or spliced genes. The method was performed using Fisher's exact test with robust FDR correction to obtain an adjusted *p* value between certain tested gene groups and the whole annotation. SignalP v. 4.1 (Nielsen, 2017) was used to predict N‐terminal secretion signals of reference and novel isoforms. Transmembrane domains were predicted with TMHMM v. 2.0 (Krogh et al., 2001).

### 4.5 RT‐PCR

RNA was collected as for the RNA‐seq experiment. Reverse transcription was performed using 0.5 µl of SuperScript II reverse transcriptase (Invitrogen), 1 µg of oligo(dT), 10 nmol of deoxynucleotide triphosphate (dNTP), and 1 µg of total RNA in a 20 µl reaction. RNA samples collected from three plants of each species were pooled together for cDNA synthesis. RT‐PCR was performed using gene‐specific primers (Table S8) on an Eppendorf G‐storm GS2 Mastercycler with PCR conditions 4 min at 94°C, followed by 32 cycles of 30 s at 94°C, 30 s at 55°C, and 1 min at 72°C, followed by 10 min at 72°C.

### 4.6 Protein 3D structure modelling and visualization

Protein structure models were determined using the I‐TASSER online server (Yang et al., 2015). Top protein models retrieved from I‐TASSER searches were rendered using the UCSF Chimera v. 1.11.2 software. Models were superimposed using the MatchMaker function in Chimera, best‐aligning pairs of chains with the Needleman–Wunsch algorithm with BLOSUM‐62 matrix and iterating by pruning atom pairs until no pair exceeds 2.0 Å.

## AUTHOR CONTRIBUTIONS

H.M.M.I. suggested the idea, designed the experiment, performed the bioinformatics analyses, and drafted the manuscript. S.K. contributed to the bioinformatics analyses and drafted the manuscript. M.D. performed the RT‐PCR. S.R. monitored the RT‐PCR experiment, contributed to bioinformatics analyses, revised the manuscript, and provided feedback. All authors edited, proofread, and approved the final version of this manuscript.

## Data Availability

All data sets generated for this study are included in the manuscript and the supplementary files. RNA‐Seq data are deposited in the NCBI Gene Expression Omnibus (GEO) repository at https://www.ncbi.nlm.nih.gov/geo/ under accessions GSE106811, GSE116194, and GSE138039.
