# Supplementary figures and images for: Genome‐wide alternative splicing profiling in the fungal plant pathogen Sclerotinia sclerotiorum during the colonization of diverse host families
Source: Mol Plant Pathol. 2020 Oct 28;22(1):31–47. doi: 10.1111/mpp.13006 (PMC7749757; doi:10.1111/mpp.13006)

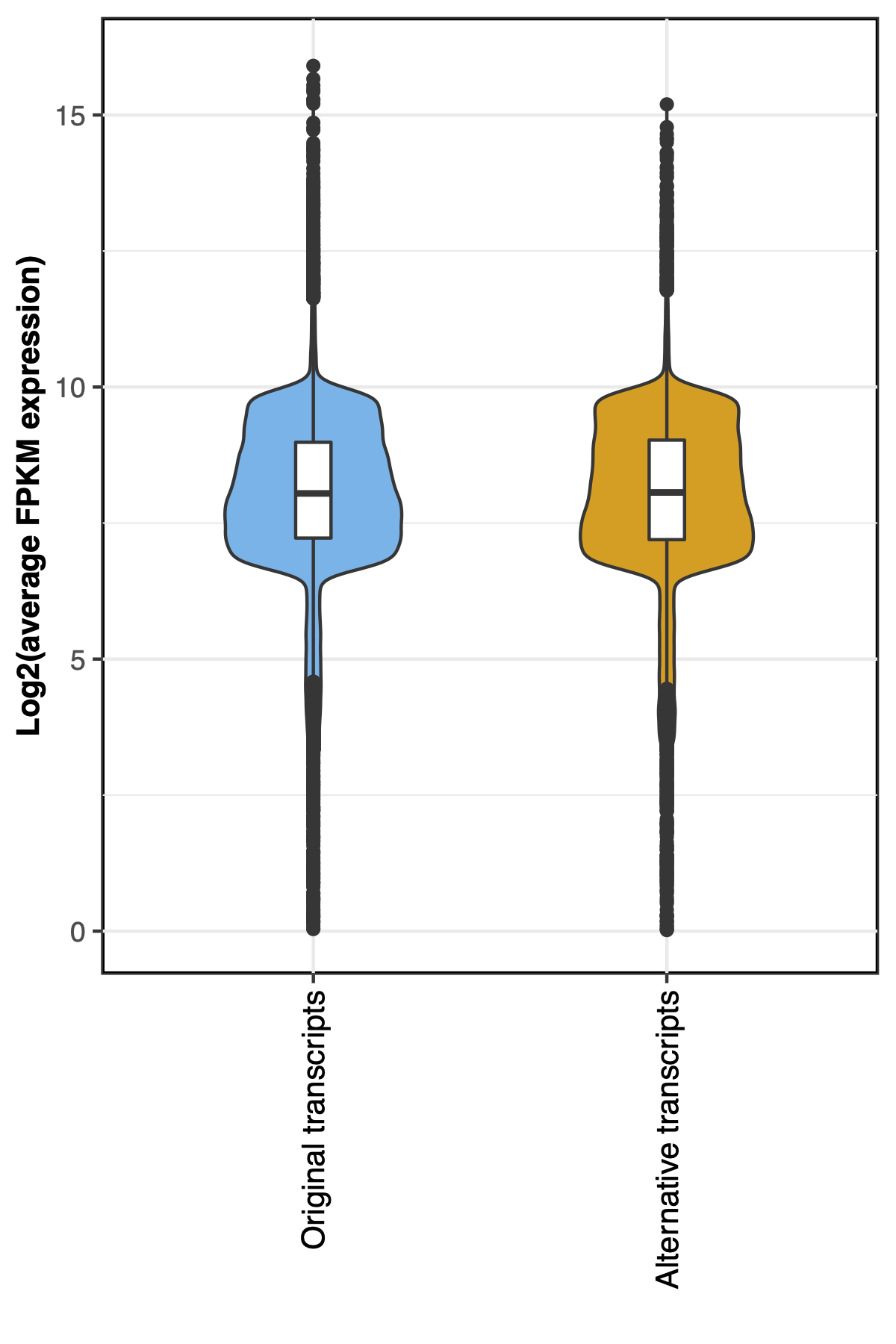

Supplement: Supplementary file 1 [file MPP-22-31-s001.jpg]

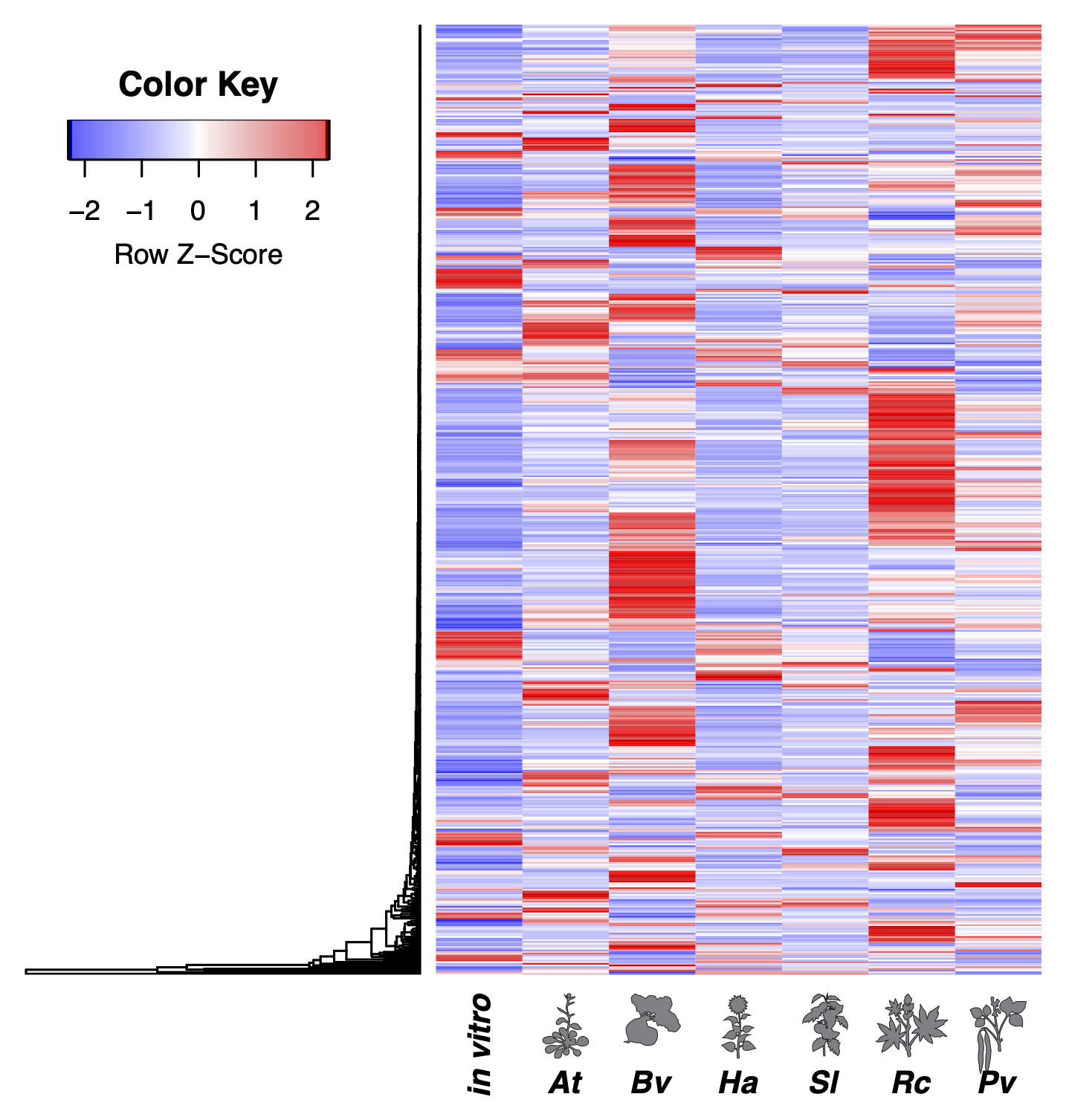

Supplement: Supplementary file 2 [file MPP-22-31-s002.jpg]

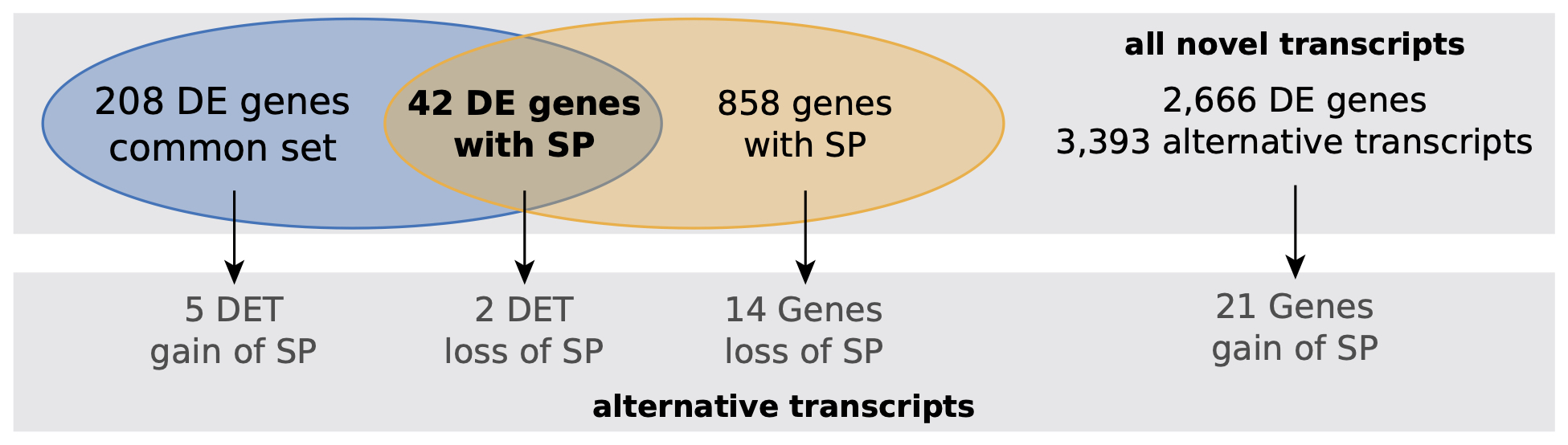

Supplement: Supplementary file 3 [file MPP-22-31-s003.jpg]
